# Oral emergency contraception practices of community pharmacies: a mystery caller study in the capital of Germany, Berlin

**DOI:** 10.1186/s40545-023-00565-w

**Published:** 2023-05-26

**Authors:** Gwenda Lungfiel, Franca Mandlmeier, Christian Kunow, Bernhard Langer

**Affiliations:** Department of Health, Nursing, Management, University of Applied Sciences Neubrandenburg, Neubrandenburg, Germany

**Keywords:** Non-prescription medicines, Emergency contraception, Community pharmacies, Counseling, Availability, Pricing, Mystery calls, Germany

## Abstract

**Background:**

In Germany, oral emergency contraception (EC) with the active ingredients levonorgestrel (LNG) and ulipristal acetate (UPA) is available as over-the-counter (OTC) medicine only from community pharmacies (CPs). Because of the window of effect, which is limited to only a few days, CPs have a great responsibility to provide rapid and unimpeded access, while also ensuring “adequate” counseling. The aim was—for the first time in Europe and thus also in Germany for the methodology used in this study—to investigate immediate availability, pricing, and aspects of counseling.

**Methods:**

Covert mystery calls were conducted in a random sample of CPs stratified by districts in the German capital Berlin. Each of the 263 CPs included was called once at random by one of two trained female student mystery callers. They simulated a product-based scenario for the UPA original ellaOne^®^, citing a contraceptive failure one day ago as the reason.

**Results:**

Of 257 successfully called CPs, UPA preparations were immediately available in 98.4% (253/257) and LNG preparations in 86.8% (184/212) of CPs. Prices for UPA preparations varied from €15.95 to €42.95 (∆ 169%; median €35.00 [interquartile range (IQR) €5.91]) and for LNG preparations from €10.60 to €32.49 (Δ 207%; median €22.00 [IQR €5.76]). Information about the correct different window of effect of UPA and LNG preparations was provided in 69.8% (127/182) of CPs. UPA preparations were recommended in 63.1% (111/176) and LNG preparations in 17.2% (30/174) of CPs. Information was provided on how to take them as soon as possible in 30.8% (44/143) of CPs and on how to use them after vomiting in 46.0% (64/139).

**Conclusions:**

Berlin CPs support access through high immediate availability, especially to UPA preparations. However, access is hampered by high absolute price ranges of both UPA and LNG preparations, which could ideally be minimized by a comparison app. It is positive that CPs promote the benefits of UPA preparations by recommending them noticeably more often than LNG preparations. However, there are deficiencies in giving advice, so there is a need to raise awareness among pharmacy staff to ensure “adequate” counseling in advance over the phone.

**Supplementary Information:**

The online version contains supplementary material available at 10.1186/s40545-023-00565-w.

## Background

To avoid an unwanted pregnancy due to unprotected sexual intercourse (UPSI), e.g., contraceptive failure, the World Health Organization recommends the use of emergency contraception (EC) [1]. The best known EC in Germany is the “morning after pill” (oral EC) [2]. Oral EC in the form of combined oral contraceptives (Yuzpe method) is not recommended in Germany [3]. However, oral EC is approved as a single-dose preparation, containing one of the two active ingredients levonorgestrel (LNG) and ulipristal acetate (UPA) [3]. For both, numerous generics are available in Germany in addition to the LNG original PiDaNa^®^ and the UPA original ellaOne^®^ [4]—without legal age restrictions and according to the product information of LNG and UPA preparations for all women of childbearing age [5]. LNG is effective up to 72 h after UPSI. UPA is in contrast to LNG even effective for up to 120 h after UPSI and thus effective for 48 h longer. Moreover, UPA has been shown to be more effective in the first 24 or 72 h after UPSI [6, 7]. Because of this window of effect, which is limited to only a few days, access to oral EC (especially UPA) as quickly as possible and low barriers to access are of particular importance.

In contrast to some other countries [8], oral EC is also available in Germany as an over-the-counter (OTC) medicine without prescription, i.e., for self-medication without prior consultation with a physician [9]. Of the more than 800,000 packages of oral EC dispensed each year, approximately one-third contain the active ingredient LNG and two-thirds the active ingredient UPA [10]. More than 90% of the packages are dispensed without a prescription [11]. In contrast to some other countries [12], OTC medicines in Germany may only be dispensed by community pharmacies (CPs) [13]. Compared to the USA [14], in German CPs OTC medicines, especially oral EC, are usually only available behind the counter and thus not accessible without barriers. In this respect, German CPs have a great responsibility with regard to immediate availability and pricing as important criteria for the fastest and most unhindered access to oral EC [15, 16]. In terms of availability, this is important because the faster oral EC is taken, the more effective it is [1]. In terms of pricing, it should be noted that German CPs are free to set the price of oral EC as an OTC medicine due to the lack of price control [17]. In addition, the actual prices for oral EC of the individual German CPs are not available online, which raises the question of what prices are actually charged. This lack of price transparency is one of the main reasons for possible price ranges [18].

The CPs in Germany must also ensure “adequate” counseling [19] in the sense of a multi-stage process from information gathering, possible preparation recommendations to giving advice [20]. In addition to pharmacists, this can also be done by non-pharmacists (pharmacy technicians and pharmaceutical technical assistants) if the pharmacy manager has previously specified this [19]. “Adequate” counseling is particularly important against the background that surveys have found knowledge deficits and incorrect knowledge about oral EC in adolescents [21], adults [22] and across populations [2, 23] in Germany, especially with regard to the mechanism and period of time of action. To assist pharmacy staff in counseling, the German Federal Chamber of Pharmacists (BAK) has published guidelines [5]. In addition to content on giving advice (e.g., on the correct different window of effect as well as on the intake behavior), these guidelines also contain recommendations on oral EC. An important component of the guidelines is a checklist, which the CPs should have available in their daily pharmacy routine. The aim of this checklist is to gather information to ensure that the pharmacy staff ask the patients questions that are essential for "adequate" counseling.

Unlike other countries in particular such as the USA [14–16, 24–60], the study situation for Germany for availability, pricing and counseling for oral EC is rather poor so far. It should be noted that three German studies [23, 61, 62]—in contrast to another German study [63]—are to be classified as gray literature. In all four mentioned studies, exclusively self-reported surveys and interviews with the risk of a social desirability bias were used for data collection. Therefore, the international literature recommends the use of simulated patient methodology (SPM) as a form of covert participatory observation [64, 65]. Compared to other indications [66], only one German SPM study is known for oral EC, which exclusively investigated aspects of counseling [67]. Whether giving advice on the correct different window of effect and on the intake behavior was explicitly not investigated. No German SPM study is available on possible preparation recommendations. However, at least enough is known that German community pharmacists generally declared UPA preparations as the first-line option [63]. For availability, no German SPM study is known to date either, although most German community pharmacists surveyed in another self-reported study stated that they had both UPA and LNG preparations immediately available [23]. For pricing, in fact, no single German study is known. However, at least three German SPM studies for other OTC medicines have already shown that price ranges also exist within geographically narrowly defined areas (e.g., for cities) [68–70].

Using the SPM, the primary objective of this study was therefore to investigate for oral EC:

- the immediate availability and pricing (both UPA and LNG),
- aspects of counseling such as possible preparation recommendations as well as giving advice on the correct different window of effect and on the intake behavior.

Secondarily, the potential influence of different independent factors on immediate availability, pricing, and aspects of counseling should be determined.

## Methods

### Design

This is a cross-sectional study in which the SPM was applied. According to the international literature [64, 71, 72], a seemingly real customer covertly contacts a CP to simulate a conversational situation as close to real life as possible based on a previously defined scenario. Afterwards, the data collection is carried out with the help of predefined items using an assessment form. The SPM in the form of on-site visits involves considerable time and financial effort, especially for larger sample sizes [73]. Therefore, the SPM in the form of mystery calls, which has been frequently used in a CP setting [15, 24–26, 28–34, 36–60, 74, 75], is suitable. In this context, so-called mystery callers (MCs) simulate the conversational situation on the phone.

This SPM study was reported according to the STROBE Statement [76] and, based on this, according to the guidelines for health care simulation research specific to SPM [77] and the “Checklist for Reporting Research Using Simulated Patient Methodology” (CRiSP) [78].

### Mystery caller

The mystery calls of this SPM study were performed by two MCs (GL and FM) as female master’s students of the Department of Health, Nursing, Management of the University of Applied Sciences Neubrandenburg as part of their master thesis free of charge. Both MCs are of German ethnicity and were 26 and 28 years old, respectively, when the study took place. Both students had no previous experience as MCs, but acted as simulated patients (SPs) in 2019 as part of a SPM visit study [67].

### Setting and participation

From October 19 to November 4, 2020, the MCs called CPs of the German capital Berlin, which is by far the largest German city (December 31, 2020: approx. 3.66 million residents; approx. 891 km^2^ area; high population density of approx. 4112 residents/km^2^ [79]). First, all CPs and the relevant information (name, postcode, phone numbers, opening hours) stored in the online pharmacy finder of the Berlin Chamber of Pharmacists as of the cut-off date of August 15, 2020 [80] were extracted, with the search engine Google being used to help in the case of missing information. Then, the 769 CPs included in this process were stratified according to the 12 Berlin districts that differ in the purchasing power of their households [81].

Since there were no studies on the subject of the study in Germany, the proportion of the characteristic of interest in the population was unknown. The minimum required sample (*n*) was determined for the corresponding population size (*N*) and an error margin (*e*) of 0.05 using the following formula, which was based on an assumed proportion of the characteristic of interest in the population of *P* = 0.5 and on a 95% confidence level [82]:

$$n= \frac{N}{1+N{(e)}^{2}}=\frac{769}{1+769{(0.05)}^{2}}=263$$

$$Population\; size=N | Error\; margin=e.$$

The assumed proportion of the characteristic of interest in the population of *P* = 0.5 maximized the required sample. The 769 CPs stratified by the 12 Berlin districts were randomly numbered using the random number function of Microsoft^®^ Excel version 16.45 (Microsoft Corporation, Redmond, USA). A simple random sample was then drawn in each stratum to the extent of that stratum’s proportion of all CPs to achieve the required 263 CPs. The 263 CPs were randomly distributed among the MCs, with each of the two MCs being allocated nearly equal numbers of CPs, 131 and 132 CPs, respectively. Each of the CPs should be successfully called once, so that there would be a total of 263 successful mystery calls (one MC × 131 CP + one MC × 132 CP = 263 CPs = 263 mystery calls).

One month before the main study with the planned 263 mystery calls, both MCs conducted eight validation mystery calls each (16 in total) in the context of a pilot study with Berlin CPs also randomly selected outside the sample. On the one hand, MCs should train the SPM in practical application. On the other hand, the functionality of the scenario and the assessment form should be tested in practice, whereby no changes were necessary.

### Scenario and assessment

**Fig. 1:**
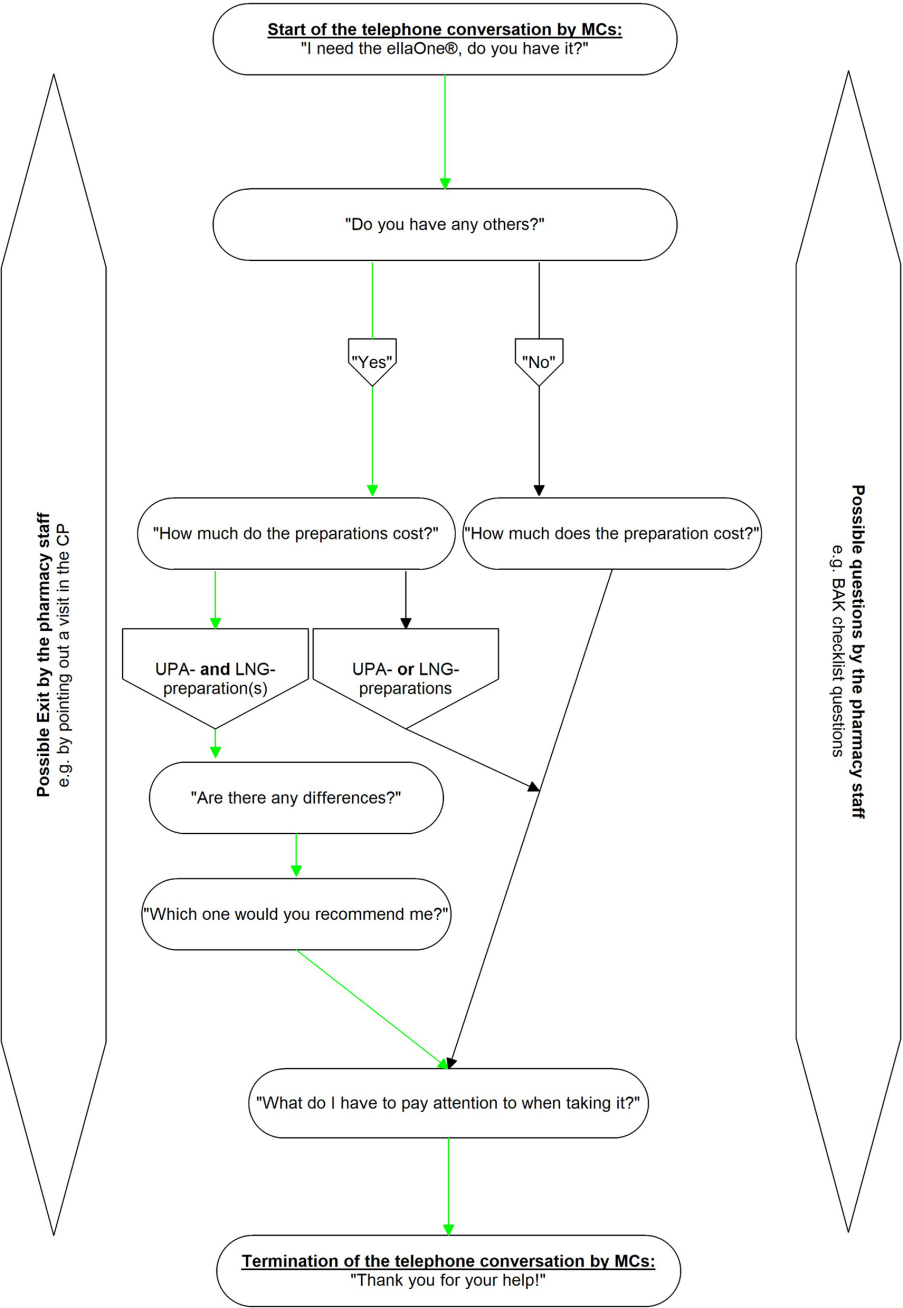
Scenario for MCs using a flowchart. The green arrows indicate the most optimal course of conversation

As a refresher, both MCs first familiarized themselves with the theoretical basics of SPM. Then, based on the guidelines of the BAK [5], they developed the scenario (Fig. 1) and the assessment form (Table 1), whose items were based on the questions of the MCs of the scenario. Before the practical testing of the functionality, the scenario and the assessment form were first subjected to a content validation by BL, a researcher experienced in the application of the SPM in a CP setting. This also revealed no need for change.

**Table 1:**
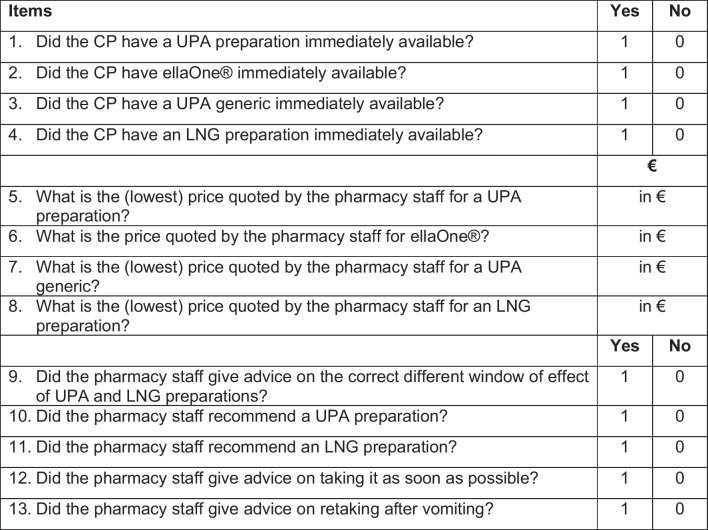
Assessment form

The items are listed in their order according to the planned scenario

The respective conversational situation was based on a product-based scenario. The reason for a product-based rather than a symptom-based scenario was that the immediate availability and pricing of UPA and LNG preparations as well as aspects of counseling should be investigated, and the pharmacy staff should be specifically directed to this. The scenario was designed as a "normal scenario" so that the limits of self-medication (e.g., the (immediate) recommendation of a physician visit) should not be exceeded. In each section of the scenario, a possible termination of the conversation (exit) on the part of the pharmacy staff was planned as a precaution, e.g., by referring to a visit in the CP. The scenario basically provided for an active role of the MCs, according to which they should ask the pharmacy staff questions. The questions were predefined in such a way that they were quite easy for the MCs to simulate and the possible answers should be quite uncomplicated for the pharmacy staff.

At the beginning of the scenario, the MC should ask the pharmacy staff who answered the mystery call whether the UPA preparation ellaOne^®^ required for herself was immediately available. The reason for selecting a UPA preparation was that they are superior to LNG preparations [6, 7] and were also considered more appropriate by community pharmacists in a fairly recent German study [63] (item 1 of the assessment form). The question specifically about the UPA original ellaOne^®^ was set because it is probably better known in the population than the term “ulipristal acetate” or its abbreviation “UPA” and probably also than the trade names of UPA generics (item 2 of the assessment form). After the onset, the scenario included a question about other preparations of oral EC, which was intended to assess both the immediate availability of UPA generics (item 3 of the assessment form) and LNG preparations (item 4 of the assessment form). For the preparations discussed up to this point, the respective prices were requested in order to evaluate them (items 5–8 of the assessment form). If at least one UPA and at least one LNG preparation had been discussed by then, the question about the difference should be asked in order to evaluate whether the pharmacy staff were giving advice on the correct different window of effect of LNG and UPA preparations (item 9 of the assessment form). This was immediately followed by the question of which preparation (UPA or LNG or both) is recommended, which was also included in the assessment (items 10 and 11 of the assessment form). At the end of the scenario, the question should be asked what to look for when taking the medication. This was used to assess whether the pharmacy staff had given advice on taking the medication as quickly as possible due to the window of effect being limited to only a few days (item 12 of the assessment form) and on taking the medication again after vomiting due to the possible reduction in efficacy (item 13 of the assessment form).

In the case of possible questions of the pharmacy staff for information gathering based on the questions of the checklist of the BAK [5], standardized answers were provided for the MCs (Table 2). This should prevent different preparations being recommended due to divergent answers. The answers were predefined in such a way that on the one hand, they were quite easy to simulate for the MCs. On the other hand, the information gathering for the pharmacy staff should be quite uncomplicated. The indication of the real age of the MCs to the corresponding question resulted in a realistic representation of the MCs’ voice. The answer to the question about the reason for needing oral EC/ellaOne^®^ was quite close to reality, as contraceptive failure (e.g., due to a broken condom or a forgotten use of regular contraception) was mentioned most frequently by both surveyed girls and young women [83] and surveyed community pharmacists [63] in two quite recent German studies. Also in the latter study, the time interval between UPSI and contact with a CP was cited as the most important criterion for selecting an oral EC [63]. Therefore, the answer “One day ago”. to the question about the timing of UPSI/contraceptive failure was intended to support the simulation of a “normal scenario”, potentially allowing the recommendation of both UPA and LNG preparations, thereby achieving the study objectives. Finally, the answer to the question about the time of the last menstrual period should also give an indication of potential efficacy in a possible preparation recommendation. The “No” response to the other possible checklist questions was also intended to support the simulation of a “normal scenario”. To unplanned questions from the pharmacy staff, MCs should respond with “I don’t know.” and not respond at all to comments made by the pharmacy staff in order to simulate the scenario as much as possible as planned.

**Table 2:** Possible questions by the pharmacy staff and response specifications for MCs

Possible questions by the pharmacy staff for information gathering based on the questions of the BAK checklist [5]	Response specifications for MCs
“How old are you?”	“I am [real age of the MCs].”
“Why do you need oral EC/ellaOne®?”	“We had a contraceptive failure.”
“When was the unprotected sexual intercourse/ contraceptive failure?”	“One day ago.”
“When was the last menstrual period?”	“11 days ago.”
“Is the date of the first day of the last menstrual period more than 28 days ago?”	“No”
“Was the last menstrual period weaker than usual?”	“No”
“Was the last menstrual period shorter than usual?”	“No”
“Was the last menstrual period unusual in any other way?”	“No”
“Are you aware of any acute health problems or chronic illnesses?”	“No”
“Are you currently breastfeeding?”	“No”
“Are you currently taking any medication?”	“No”
“Have you ever used oral EC before?”	“No”

In addition to the items of the assessment form, the MCs before and during the mystery calls also collected numerous factors that may have an influence on the assessment items, analogous to the international literature (Table 3).

**Table 3:** Possible influencing factors, time and type of data collection

Possible influencing factors [Literature sources*]	Time of data collection	Type of data collection
Districts of the CPs as an indicator of lower-income/higher-income [26]	Before the mystery call	Exact measurement by assigning the number of determined CPs to the respective districts
Time of the mystery call [75]	During the mystery call	Exact measurement using the MCs’ watch
Gender of the pharmacy staff [74]	During the mystery call	Estimate by acoustic impression of the MC
Call attempts [52]	During the mystery call(s)	Exact measurement by number of call attempts
Forwarding to other staff [42]	During the mystery call	Exact measurement by acoustic impression of the MC
Call duration [44]	During the mystery call	Exact measurement based on the display of the phone of the MC
Placed on hold after call acceptance [47]	During the mystery call	Exact measurement by calculating the start and end point of the waiting time using the MCs’ watch

*The possible influencing factors were taken from the specific literature sources

### Data collection

The data collection based on the mystery calls took place on different days of the week and at different times of the day within the opening hours of the CPs. Each CP was first called once. If the CP could not be reached, a second and, if necessary, a maximum of a third call attempt was made. All attempts took place on the same day, at five to ten minutes intervals, in order to simulate as realistic a situation as possible, especially for such an emergency. Thus, it seems realistic that affected persons call the next CP after a certain point in time or after a certain number of attempts. After the third attempt, the CP was noted as “not reachable”. For the most realistic depiction, MCs did not explicitly ask for a pharmacist, but instead conducted the conversation with the pharmacy staff who were on the phone. This was to determine the extent to which the pharmacy staff of each CP is trained regarding oral EC and can provide appropriate giving advice. In addition, no phone number transmission took place. This should avoid that called CPs could call back or that CPs still to be called could be forewarned. At the end of the mystery calls, the MCs did not reveal themselves and completed the assessment form in writing immediately following the mystery calls with subsequent transfer to a digital form. Since CPs in Germany are classified as system-relevant and must therefore remain accessible [84], the COVID-19 pandemic should not have had any influence on the implementation of the data collection.

### Data management and analysis

Data were entered using the four-eyes principle and analyzed using SPSS version 27 for Windows (IBM, Armonk, NY, USA). Descriptive statistics were used to determine frequencies and percentages for the categorical data. The Chi-square test (or alternatively, for expected cell frequencies less than five, the Fisher [85] or Fisher–Freeman–Halton [86] exact test) was used to identify possible associations between each of the assessment form categorical variables (Table 1) and the potential influencing factors (Table 3). Cramer’s *V* was reported as the effect size measure. For significant results of Chi-square and Fisher–Freeman–Halton’s exact tests for contingency tables larger than 2 × 2, post hoc analyses with z-tests for independent proportions using a Benjamini–Hochberg adjustment for multiple comparisons were performed. Application of the Shapiro–Wilk test revealed that the continuous price variables of UPA and LNG preparations are not normally distributed. Therefore, for these variables median, interquartile range (IQR), min., max., and range were calculated. In addition, to determine price differences with respect to the possible influencing factors, the non-parametric Kruskal–Wallis (analogous to [85], reporting of an effect size with more than one degree of freedom was omitted) and Mann–Whitney *U* (effect size Pearson’s *r*) tests for independent samples were performed. In all statistical analyses, a *p*-value less than 0.05 was considered significant.

### Performance feedback

Following the data analysis, each CP received—as recommended internationally [71, 72]—a written (by mail and if not possible by letter post), pharmacy-specific performance feedback using graphically prepared benchmarking (for an example see Additional file 1). In this way, the CPs gained knowledge about their competitive position with regard to the individual assessment items. Both a Berlin-wide and a district-wide comparison were made with the remaining CPs of the stratified random sample, which were presented anonymously. The aim was to stimulate optimization processes on the part of the CPs in order to sustainably increase the quality and safety of care.

### Ethical approval

The collected data were recorded anonymously according to the “Guideline for the Use of Mystery Research in Market and Social Research” [87] and processed in such a way that the pharmacy staff of the individual CPs could not be identified. Analogous to the international literature [71], mystery calls were conducted covertly (without prior information to CPs) to avoid both a “Hawthorne effect” [88] and a selection bias [89]. This meant that informed consent could not be obtained in advance from individual CPs to participate. The lack of informed consent in advance was addressed, analogous to the international literature [90], by informing all CPs of the background and conduct of the study three months after all mystery calls were completed. No complaints or comments were received about the SPM after informing about the study (and providing feedback) to the CPs. Recruited students provided their written informed consent to act as MCs. The study protocol was approved by the institutional ethics committee of the Neubrandenburg University of Applied Sciences (registration number: HSNB/168/20). Non-disclosure to participants was considered ethically acceptable by the ethics committee, as the data were kept anonymous and none of the mystery calls were audiotaped.

## Results

No costs were incurred for making the mystery calls, as both MCs had a phone flat rate and the corresponding monthly basic charges for the MCs were incurred anyway. As planned, an attempt was made to successfully call each of the 263 included CPs once. However, six CPs (2.3%) could not be reached even after the third call attempt, so that ultimately 257 CPs/mystery calls could be taken into account (call completion rate: 97.7%).

The characteristics of the CPs, pharmacy staff, and mystery calls are shown in Table 4. The majority of mystery calls were made between 12:01 pm and 4:00 pm (40.9%, 105/257), and the interlocutors were mostly female (84.8%, 218/257). Most CPs (87.2%, 224/257) could be reached on the first call attempt. In relatively few cases (9.3%, 24/257) the mystery calls were forwarded to other pharmacy staff. With regard to the duration of the mystery calls, there was a very wide range from 0:31 min to 8:33 min. In most cases (86.4%, 222/257) the MCs did not have to accept a place on hold after call acceptance.

**Table 4:** Characteristics of CPs, pharmacy staff, and mystery calls

	Frequency (*n*)	Percentage (%)
All CPs/mystery calls	257	100
Districts of the CPs		
Charlottenburg-Wilmersdorf	35	13.6
Friedrichshain-Kreuzberg	19	7.4
Lichtenberg	16	6.2
Marzahn-Hellersdorf	17	6.6
Mitte	28	10.9
Neukölln	20	7.8
Pankow	24	9.4
Reinickendorf	14	5.4
Spandau	15	5.8
Steglitz-Zehlendorf	24	9.4
Tempelhof-Schöneberg	28	10.9
Treptow-Köpenick	17	6.6
Time of the mystery call		
08:00 am –12:00 pm	78	30.3
12:01 pm – 04:00 pm	105	40.9
04:01 pm–08:00 pm	74	28.8
Gender of the pharmacy staff		
Male	39	15.2
Female	218	84.8
Call attempts		
1 attempt	224	87.2
2 attempts	26	10.1
3 attempts	7	2.7
Forwarding to other staff		
No	233	90.7
Yes	24	9.3
Call duration		
0:00–1:00 min	48	18.7
1:01–2:00 min	69	26.8
2:01–3:00 min	68	26.5
3:01–4:00 min	43	16.7
> 4:00 min	29	11.3
Placed on hold after call acceptance		
None	222	86.4
≤ 1:00 min	33	12.8
> 1:00 min	2	0.8

During the mystery calls, some of the interlocutors ended the conversation prematurely (exit), so that not all items of the assessment form were available for all 257 CPs/mystery calls. This resulted in different or decreasing sample sizes for items 1–13 (Table 5).

**Table 5:** Assessment items

	Total (*n*)	Frequency (*n*)	Percentage (%)
1. Immediate availability of a UPA preparation	257	253	98.4
2. Immediate availability of ellaOne®	253	241	95.3
3. Immediate availability of a UPA generic	253	24	9.5
4. Immediate availability of an LNG preparation	212	184	86.8

	Total (*n*)	Median [IQR] (EUR)	Range (EUR)
5. (Lowest) price of UPA preparations	241	35.00 [5.91]	15.95–42.95
6. Price of ellaOne^®^	224	35.07 [2.07]	25.50–42.95
7. (Lowest) price of UPA generics	50	24.90 [1.38]	15.95–32.00
8. (Lowest) price of LNG preparations	160	22.00 [5.76]	10.60–32.49

	Total (*n*)	Frequency (*n*)	Percentage (%)
9. Giving advice on the correct different window of effect	182	127	69.8
10. Recommendation of a UPA preparation	176	111	63.1
11. Recommendation of an LNG preparation	174	30	17.2
12. Giving advice on taking as soon as possible	143	44	30.8
13. Giving advice on retaking after vomiting	139	64	46.0

UPA preparations were immediately available in 98.4% (253/257) of CPs, which means that in 1.6% (4/257) no UPA preparation was immediately available. Among these, immediate availability of the UPA original ellaOne^®^ was present in 95.3% (241/253) of CPs. 9.5% (24/253) of CPs indicated immediate availability of a generic UPA product, and 4.7% (12/253) had only a generic UPA product immediately available. Immediate availability of LNG preparations was evident in 86.8% (184/212) of CPs; conversely, 13.2% (28/212) had no LNG preparations immediately available. Across preparations, one CP (1/212) had neither a UPA nor an LNG preparation immediately available.

The median price for UPA preparations (ellaOne® and generics) (*n* = 241) was €35.00 [IQR €5.91] with a price range from €15.95 to €42.95 (Δ €27.00; 169%). The UPA original ellaOne^®^ (*n* = 224) had a median price of €35.07 [IQR €2.07] with a price range from €25.50 to €42.95 (Δ €17.45; 68%) and UPA generics (*n* = 50) had a median price of €24.90 [IQR €1.38] with a price range from €15.95 to €32.00 (Δ €16.05; 101%). For LNG preparations (*n* = 164), the median price was €22.00 [IQR €5.76] with a price range from €10.60 to €32.49 (Δ €21.89; 207%). Thus, the median price for UPA preparations was 59.1% higher than the median price for LNG preparations. Prices for Berlin districts are reported in Additional file 2.

69.8% (127/182), and thus the majority of CPs, provided information about the correct different window of effect of UPA and LNG preparations. UPA preparations were recommended far more frequently (63.1%, 111/176) than LNG preparations (17.2%, 30/174). In 30.8% (44/143), information was given about taking the drug as soon as possible and in 46.0% (64/139) about retaking after vomiting.

There were no significant associations between the availability and prices of UPA and LNG preparations (items 1–8 of the assessment form) and the possible influencing factors. Table 6 shows the associations between the aspects of counseling (items 9–13 of the assessment form) and the possible influencing factors. A significant association was found between “call duration” and “giving advice on the correct different window of effect” (Fisher–Freeman–Halton exact test; *p* = 0.002, *V* = 0.309), with an effect size corresponding to a “medium” effect according to Cohen [91]**.** In post hoc analyses, a call duration between 2:01 and 3:00 min (Benjamini–Hochberg adjusted *p* = 0.044), between 3:01 and 4:00 min (Benjamini–Hochberg adjusted *p* = 0.010), or above 4:00 min (Benjamini–Hochberg adjusted *p* = 0.010) compared to 1:01 to 2:00 min were significantly associated with increased “giving advice on the correct different window of effect”. Similarly, the “call duration” and the “giving advice on retaking after vomiting” (Fisher–Freeman–Halton exact test; *p* = 0.001, *V* = 0.362) showed a significant association, which also had a “medium” effect size [91]. Post hoc analyses showed that a call duration between 3:01 and 4:00 min (Benjamini–Hochberg adjusted *p* = 0.012) or above 4:00 min (Benjamini–Hochberg adjusted *p* = 0.012) compared to 1:01 to 2:00 min were significantly associated with increased “giving advice on retaking after vomiting”. For “giving advice on retaking after vomiting”, there was also a significant association with “districts of the CPs” (Fisher–Freeman–Halton exact test; *p* = 0.004, *V* = 0.434), also with a “medium” effect [91]. In post hoc analyses, no significant associations were found. In addition, “giving advice on retaking after vomiting” and “forwarding to other staff” showed a significant association (Pearson Chi-square test; χ^2^ [1] = 6.103, *p* = 0.013, *V* = 0.210), although the effect size was considered “small” [91].

**Table 6:** Associations between counseling items and possible influencing factors

Possible influencing factors Counseling items	*n*; *p*-value (effect size)						
	Districts of the CPs	Time of the mystery call	Gender of the pharmacy staff	Call attempts	Forwarding to other staff	Call duration	Placed on hold after call acceptance
9. Giving advice on the correct different window of effect	182; 0.238^b^ (0.278)	182; 0.994^a^ (0.008)	182; 0.148^a^ (0.107)	182; 0.749^b^ (0.085)	182; 0.861^a^ (0.013)	182; 0.002^b^* (0.309)	182; 1.000^b^ (0.070)
10. Recommendation of a UPA preparation	176; 0.126^b^ (0.306)	176; 0.274^a^ (0.121)	176; 0.632^a^ (0.036)	176; 0.103^b^ (0.144)	176; 0.240^a^ (0.089)	176; 0.996^a^ (0.033)	176; 0.951^a^ (0.005)
11. Recommendation of an LNG preparation	174; 0.271^b^ (0.282)	174; 0.481^a^ (0.092)	174; 0.385^a^ (0.066)	174; 0.456^b^ (0.118)	174; 0.205^c^ (0.117)	174; 0.505^b^ (0.135)	174; 0.380^c^ (0.094)
12. Giving advice on taking as soon as possible	143; 0.398^b^ (0.281)	143; 0.284^a^ (0.133)	143; 0.159^a^ (0.118)	143; 0.479^b^ (0.104)	143; 1.000^c^ (0.004)	143; 0.759^b^ (0.116)	143; 0.097^b^ (0.176)
13. Giving advice on retaking after vomiting	139; 0.004^b^* (0.434)	139; 0.082^a^ (0.190)	139; 0.269^a^ (0.094)	139; 0.294^b^ (0.137)	139; 0.013^a^* (0.210)	139; 0.001^b^* (0.362)	139; 0.325^b^ (0.121)

^a^*n*; Chi-square test *p*-value (Cramer’s V);

^b^*n*; Fisher–Freeman–Halton exact test *p*-value (Cramer’s V);

^c^*n*; Fisher exact test *p*-value (Cramer’s V);

*significant at *p* < 0.05

## Discussion

### Availability

In line with a German self-reported study [23], the CPs studied here quite often had preparations immediately available, with the immediate availability of UPA preparations being higher compared to LNG preparations. In contrast, it is known from international MC studies that UPA preparations in CPs in (large) cities or metropolitan areas of the USA—but as prescription-only medicine (POM)—were noticeably less immediately available (9.5% [39], 9.6% [41], 7.3% [45], 8.3% [46], 6.2% [52], 18.3% [56]). Instead, the immediate availability of LNG preparations in MC studies in (large) cities or metropolitan areas of the USA (80.0% [39], 81.5% [41], 77.6% [45], 81.9% [46], 81.2% [52], 83.2% [55]) was at a similarly high or only slightly lower level than in the present MC study. The better immediate availability of UPA preparations compared to LNG preparations may have an economic background for the German CPs due to the higher prices that can be achieved and thus presumably higher profit margins [92]. Nevertheless, high immediate availability of UPA preparations is to be welcomed due to their generally greater efficacy and longer window of effect [6, 7]. The basically quite high immediate availability of both UPA and LNG preparations can be explained by an above-average demand due to the metropolitan character and the world-famous party scene [93] of the study site Berlin, whereas most international SPM studies—with few exceptions [16]—have not identified significant differences between rural and urban CPs [15, 47, 49, 54]. However, the physical contact restrictions in place during the mystery calls due to the COVID-19 pandemic especially for the young population considered as the main users of oral EC [22] might have biased the results in different directions (lower demand or lower orders of CPs at wholesale). Nevertheless, in the present MC study, a few CPs did not have a single UPA or LNG preparation immediately available. One CP even had neither a UPA nor an LNG preparation immediately available, which is problematic considering the factor of “time” for oral EC to be taken and effective. CPs could order the preparation that is not immediately available and also have it delivered on the same day, thus enabling at least same-day availability—as some MC studies in (large) cities or metropolitan areas in the USA have investigated [24–26, 29, 38, 40, 42–44, 53, 59, 60]. However, the hours “lost” in this process could be crucial in preventing an unwanted pregnancy. This circumstance can be avoided by requiring CPs by law—as already proposed nationally [67] and internationally [51, 94, 95]—to maintain a certain stock of oral EC preparations, at least of UPA preparations.

### Pricing

In this MC study, the median price for UPA preparations was more than half higher than the median price for LNG preparations. Although comparative studies from Germany are lacking, it is known at least from international SPM studies that in the USA (UPA $50.40, LNG-original $49.93, LNG-generics $42.32; 1% and 19% difference, respectively [32, 37]) and in Turkey (UPA TL84.00, LNG TL57.00; 47% difference [96]) there is a smaller, although in Australia (UPA AU$45.00, LNG AU$20.00; 125% difference [97]) there is a larger price difference between the two preparations.

Within UPA and LNG preparations, enormous price ranges were present in this MC study, analogous to two German SPM studies on acute diarrhea. Thus, in a medium-sized city with 84 on-site visits in 21 CPs each, these two determined prices, which also included both originals and generics, from €2.36 to €8.49 (Δ €6.13, 260%) [68] and from €2.28 to €10.98 (Δ €8.70, 382%), respectively [69]. A recent German SPM study on not chronic tension-type headache in a large city with 168 on-site visits to 42 CPs even found prices also including originals and generics ranging from €0.93 to €9.97 (Δ €9.04, 972%) [70]. In contrast, the most recent international SPM studies found lower, but also in some cases even noticeably higher price ranges for oral EC, although restrictively the study sites of the following studies in Brazil and the Democratic Republic of Congo show a different price level in comparison. The Brazilian SPM study of only LNG preparations in three urban regions with 122 visits using a product-based scenario showed prices ranging from $1.25 to $5.75 (∆ $4.50, 360%) [94]. However, the Congolese SPM study also on LNG preparations in Kinshasa with 73 visits also using a product-based scenario showed prices from $0.50 to $9.20 (∆ $8.70, 1.740%) [98]. SPM studies in U.S. cities on LNG preparations showed both moderate ($34.00 to $50.00: Δ $16.00, 47% [14]) and enormous ($15.00 to $70.00: Δ $55.00, 367% [25]; $15.00 to $65.00: Δ $50.00, 333% [26]; $24.00 to $70.00: Δ $46.00, 192% [36]) price ranges. In stark contrast to all of the aforementioned studies, an MC study of UPA preparations from 10 large cities in five geographic regions across the USA identified even noticeably larger price ranges ($2.59 to $1200.99: Δ $1198.40, 46.270%) with 344 mystery calls [41].

A certain price difference between UPA and LNG preparations does not seem surprising at first, as UPA preparations are superior to LNG preparations with regard to efficacy and window of effect [6, 7]. However, the question may be asked whether this price difference seems justified—especially with regard to such an emergency situation. The rather high absolute price ranges within the UPA and LNG preparations are at first glance due to the fundamentally high absolute prices compared to medicines, for example, for acute diarrhea [68, 69] or not chronic tension-type headache [70]. In addition, however, an information asymmetry in favor of the pharmacy staff [99] as well as the very prompt self-medication required due to the emergency character and thus a rather low price elasticity of the affected persons [100] play a major role. In addition, CPs may have different profit expectations, and both customers [101] and pharmacy staff [102] may assume higher quality medicines at higher prices (e.g., ellaOne^®^ versus UPA generics). However, higher-priced medicines can represent a financial burden for patients above a certain price threshold and thus a barrier to access to oral EC, which is urgently needed at a given time.

In particular, to minimize the high absolute price ranges for oral EC and their potential consequences, price transparency should be increased [18]—despite the explicit reference by some CPs to immediately available, lower-cost UPA generics. This is particularly relevant for an on-site visit, especially since price transparency in German CPs is low due to the lack of mandatory price labeling [103] and due to price communication that usually takes place only shortly before drug dispensing [70]. Affected individuals should therefore be encouraged by public campaigns to actively inquire about and compare prices—as simulated in this MC study—and then subsequently make appropriate cost–benefit trade-offs [104]. However, this MC study showed that some CPs—despite being asked—did not provide prices (e.g., due to an exit). As part of “adequate” counseling, the pharmacy staff should therefore be sensitized to actively ask the affected individuals about their price expectations and to actively communicate the prices [105]. Ideally, those affected should be given the opportunity to make appropriate price comparisons. However, calls and especially visits on-site are quite time-consuming against the background of their time-critical emergency situation. To facilitate comparisons for those affected, it would make sense to set up a legally binding database, which already exists in Germany for the fuel prices of gas stations [106], with the current prices of the CPs. Such a database could be implemented by an app with corresponding search options such as indications, active ingredients and preferred places of purchase. Such an app also makes sense because there are noticeable price ranges even within the Berlin districts, i.e., at quite nearby CPs, which can be minimized by those affected in the sense of an efficient use of funds with a short travel time. In addition, such an app should be made known to the public through appropriate advertising measures, as only then can it exploit its full potential benefits.

### Preparation recommendations as an aspect of counseling

UPA preparations were recommended noticeably more frequently than LNG preparations in this MC study in line with a Turkish SPM study [96] and a German self-reported study [63]. This seems understandable for the scenario chosen here (UPSI one day ago), since although the BAK guidelines governing German CPs recommend LNG preparations as well as UPA preparations “equally” within 72 h after UPSI, UPA preparations are internationally attested to have greater efficacy for this time window as well [6, 7]. However, in this MC study, the possible previously asked questions of the pharmacy staff including the question about the timing of the UPSI, which is very important for a possible preparation recommendation, were not collected. It was also not collected whether the pharmacy staff were giving advice on the greater efficacy of UPA preparations within 72 h after UPSI before the possible preparation recommendation. Thus, the pharmacy staff's reason for their possible preparation recommendation remains unclear and should therefore be investigated in a further MC study using supplementary methods (questionnaires, interviews). However, it cannot be excluded that the noticeably more frequent UPA recommendation may (also) be due to the higher prices that can be achieved with UPA preparations and thus presumably higher profit margins [92]. Nevertheless, in order to maximize the efficacy of oral EC, an adjustment of the BAK guidelines to recommend only UPA even within 72 h after UPSI could be considered.

### Giving advice as an aspect of counseling

Giving advice was overall—as in international MC studies with comparable counseling items in the USA [28, 30, 34, 39, 41, 46, 49, 50, 56, 58], with few exceptions [55]—below average. While the Berlin CPs were still giving quite frequent advice on the correct different window of effect of UPA and LNG preparations, the giving advice on taking oral EC as soon as possible and about its use after vomiting were much less frequently. Especially the rather rare giving advice on the fastest possible intake is worrying, because it opens the possibility to miss the window of effect, which is limited to only a few days, and thus to increase the risk of an avoidable unwanted pregnancy. In comparison, in a recent German SPM study, also on oral EC in the federal state of Mecklenburg-Western Pomerania, with 199 on-site visits to the CPs there, giving advice on side effects was given in almost 60% of all visits—even without being asked [67]. However, the giving advice items are not directly comparable. On the other hand, it must be emphasized that counseling on the phone by the CPs may not take place to the same extent as on-site counseling. One reason could be that the pharmacy staff assume that the person concerned has to come by anyway to receive the drug and that prior counseling on the phone is therefore “unnecessary”. However, counseling on the phone can save the person a possibly avoidable trip to the CP and enable a targeted recommendation (possibly a visit to the doctor). In addition, the term “personal” counseling used in the BAK guidelines may leave room for interpretation and may also be interpreted by CPs to the effect that counseling by phone should not or need not be provided [107]. A clarifying modification of the guidelines by the BAK may therefore be useful.

### Influencing factors

Furthermore, in this MC study, certain factors had an influence on some assessment items. For example, giving advice on the correct different window of effect and the retaking after vomiting of the oral EC were significantly associated with the call duration. This could also be expected, since longer duration of conversation may also result in more giving advice. Unlike other indications [108], the SPM studies on oral EC known to the authors did not examine this possible influencing factor. The fact that giving advice on retaking after vomiting was significantly more frequent when forwarding to other staff may be explained by referral to a more knowledgeable staff. Referral was also shown to be a significant influencing factor in a U.S. MC study of oral EC, with female physicians being referred significantly more often than female and male adolescents [42]. In most SPM studies of oral EC, both study characteristics and their potential influence on assessment items tend to be understudied. In quite a few U.S. SPM studies, the influence of CP type (chain vs. independent CPs) [14–16, 25, 26, 34, 41, 44, 48, 49, 51, 52, 54, 58, 60] has been studied, although in Germany such studies are limited due to the prohibition of owning more than four CPs (max. three branch CPs allowed in addition to the main CP). In principle, in future SPM studies on oral EC, the association of as many different factors as possible with the assessment items should be examined.

### Strengths and limitations

To the authors’ knowledge, this is the first SPM study in Europe, and thus also in Germany, to examine practices of CPs on oral EC, including immediate availability, pricing as well as preparation recommendations and giving advice as aspects of counseling. The SPM in the form of mystery calls, internationally referred to as the “gold standard” [109], was applied. Here, only female MCs were used who had an age that is in the age group of the main users of oral EC in Germany [22] and thus contributed to the simulation of a lifelike conversational situation. However, there is a need for further research on whether, for example, male SPs, older SPs, SPs with a lower level of education or a different ethnicity (e.g., migrants) would be advised differently.

The study only covered the city of Berlin in the northeast of Germany, which means that the results are not transferable to other cities, especially in geographically different regions, and also not to rural regions. In addition, although the minimum sample size was slightly undercut with a subsequent continuous decrease in the sample sizes of the items of the assessment form, the results can still show a realistic picture of the practices of the Berlin CPs with regard to oral EC.

The respective mystery calls were conducted by two different MCs, which may have led to an averaging of the MCs' personal characteristics (voice) and thus to the depiction of even more realistic conversational situations. In addition, only objective items and dichotomous scales were used in order to avoid a subjective assessment and thus the MCs' leeway in assessment, which is typical for SPM studies (e.g., on the friendliness of the pharmacy staff). Nevertheless, the results of the mystery calls conducted may differ from those obtained during on-site visits. Furthermore, due to the execution of one mystery call per CP, the answers refer to only one member of the pharmacy staff. Due to the demand of the MCs explicitly for ellaOne^®^, an assessment of LNG preparations divided into original and generic products was not possible.

The inclusion in the assessment form of the possible questions for information gathering of the pharmacy staff provided for in the scenario could have enabled a fully comprehensive assessment of the counseling and also a better assessment of the respective preparation recommendation. However, due to the large number of items to be remembered, there would then have been a risk of bias in the results due to possible missing or faulty memories (recall bias) of the MCs.

No specific measures were taken to find out whether mystery callers were identified by the pharmacy staff. This would have required the willingness to cooperate of the CPs studied [110], which is likely to have been very limited due to the chosen study design (no opt-out). In addition, second observers were not used due to a lack of human resources. For reasons of data protection, no audio recordings were made either, since otherwise a corresponding consent of the selected CPs would have had to be obtained in advance, which would have enabled the CPs to refrain from participation (opt-out), which in turn would probably have led to a selection bias [89].

## Conclusions

Berlin CPs support access through high immediate availability, especially to UPA preparations. However, access is hampered by high absolute price ranges of both UPA and LNG preparations, which could ideally be minimized by a comparison app. It is positive that CPs promote the benefits of UPA preparations by recommending them noticeably more often than LNG preparations. However, there are deficiencies in giving advice, so there is a need to raise awareness among pharmacy staff to ensure "adequate" counseling in advance over the phone.

## Supplementary Information

**Additional file 1.** Example for a written, pharmacy-specific performance feedback using graphically edited benchmarking.

**Additional file 2.** Price of UPA medications (n = 241) by district, median price (minimum price - maximum price). Price of ellaOne® (n = 224) by district, median price (minimum price - maximum price). Price of UPA generics (n = 50) by district, median price (minimum price - maximum price). Price of LNG medications (n = 160) by district, median price (minimum price - maximum price).

## Data Availability

The datasets are available from the corresponding author upon reasonable request.

## Abbreviations

EC: Emergency contraception
LNG: Levonorgestrel
UPA: Ulipristal acetate
OTC: Over-the-counter
CPs: Community pharmacies
UPSI: Unprotected sexual intercourse
BAK: German Federal Chamber of Pharmacists
SPM: Simulated patient methodology
MCs: Mystery callers
CRiSP: Checklist for Reporting Research using Simulated Patient Methodology
SPs: Simulated patients
IQR: Interquartile range
POM: Prescription-only medicine
