# Supplementary material for: Oral emergency contraception practices of community pharmacies: a mystery caller study in the capital of Germany, Berlin
Source: J Pharm Policy Pract. 2023 May 26;16:68. doi: 10.1186/s40545-023-00565-w (PMC10215052; doi:10.1186/s40545-023-00565-w)
Supplement: Supplementary file 1 — Additional file 1. Example for a written, pharmacy-specific performance feedback using graphically edited benchmarking. [file 40545_2023_565_MOESM1_ESM.docx]

Additional file 1: Example for a written, pharmacy-specific performance feedback using graphically edited benchmarking


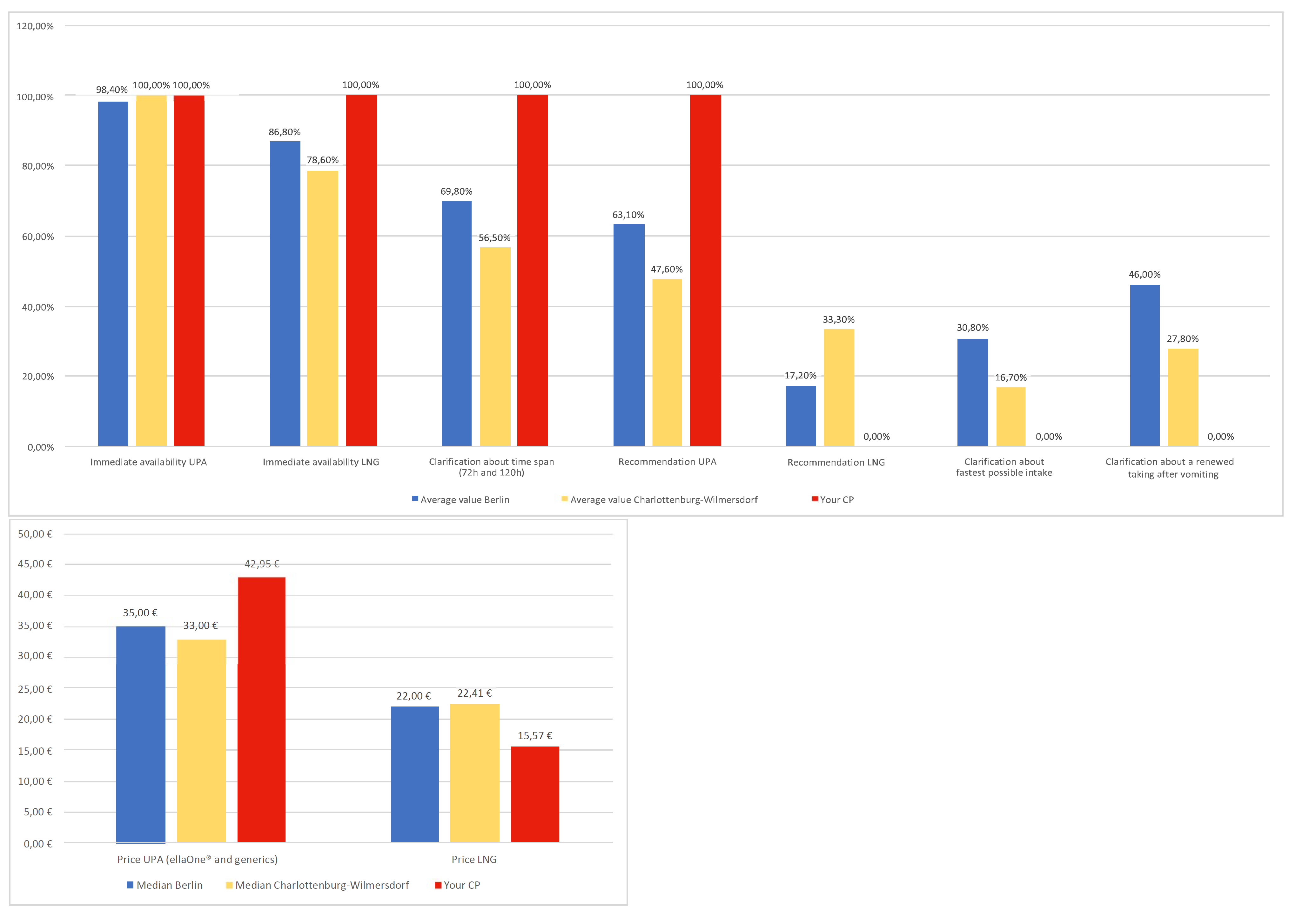


Notes:

Original in German, translated in English;

Blue bars: values for Berlin;

Yellow bars: values for the district in which the respective pharmacy is located;

Red bars: values of the respective pharmacy.
