# Supplementary material for: Oral emergency contraception practices of community pharmacies: a mystery caller study in the capital of Germany, Berlin
Source: J Pharm Policy Pract. 2023 May 26;16:68. doi: 10.1186/s40545-023-00565-w (PMC10215052; doi:10.1186/s40545-023-00565-w)

Additional file 2: Price of UPA medications (n = 241) by district, median price (minimum price - maximum price).


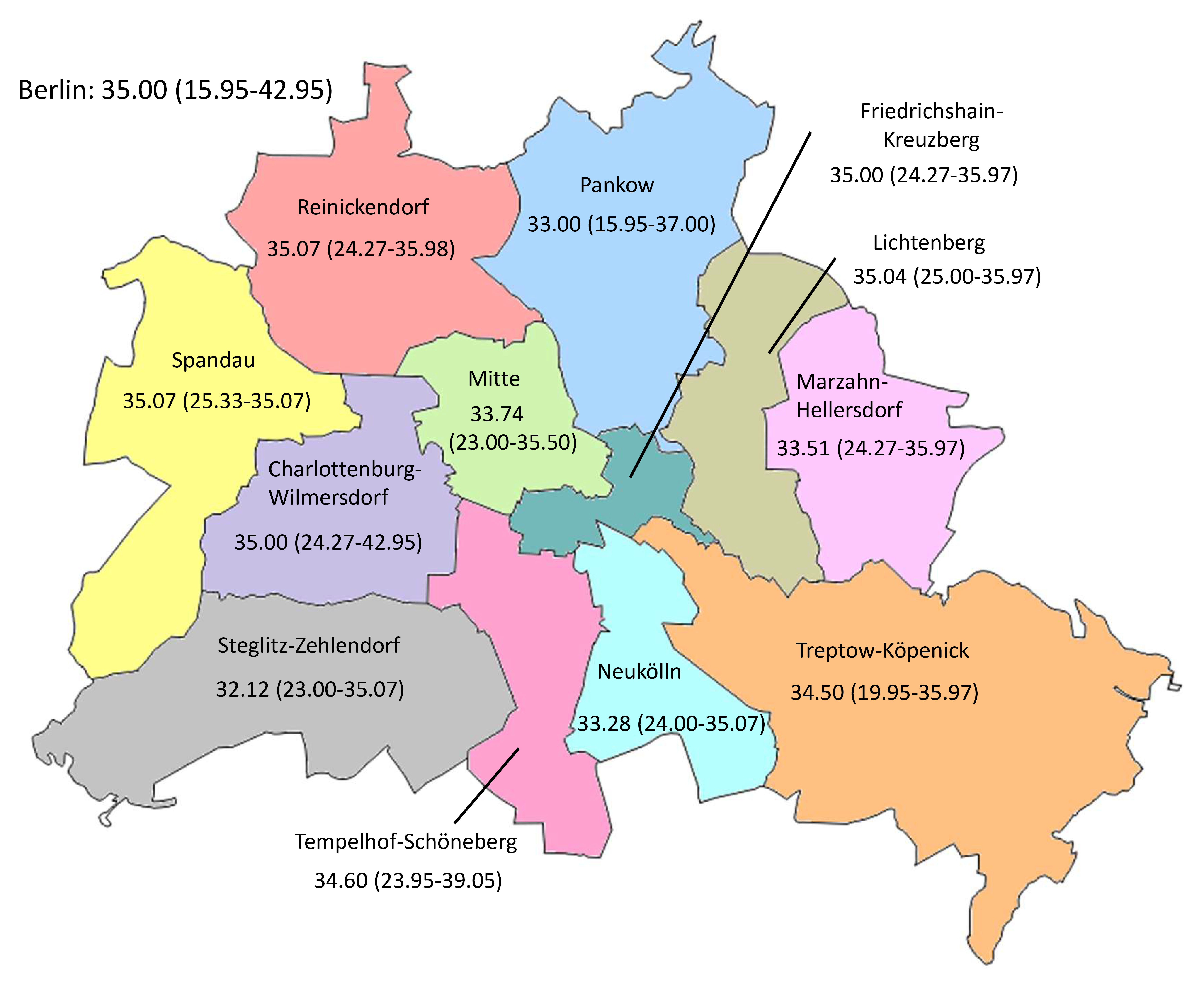


Additional file 2: Price of ellaOne® (n = 224) by district, median price (minimum price - maximum price)


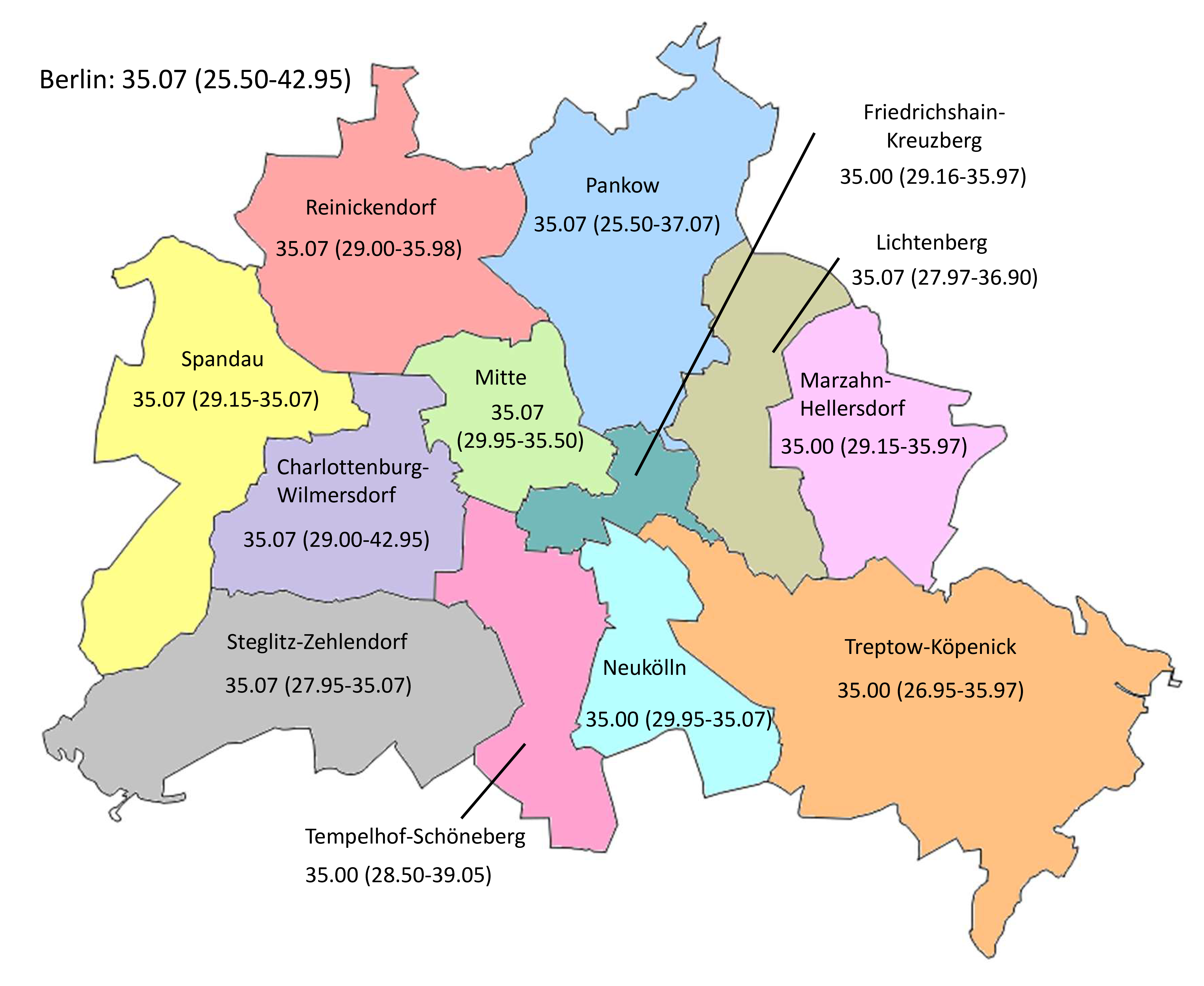


Additional file 2: Price of UPA generics (n=50) by district, median price (minimum price - maximum price).


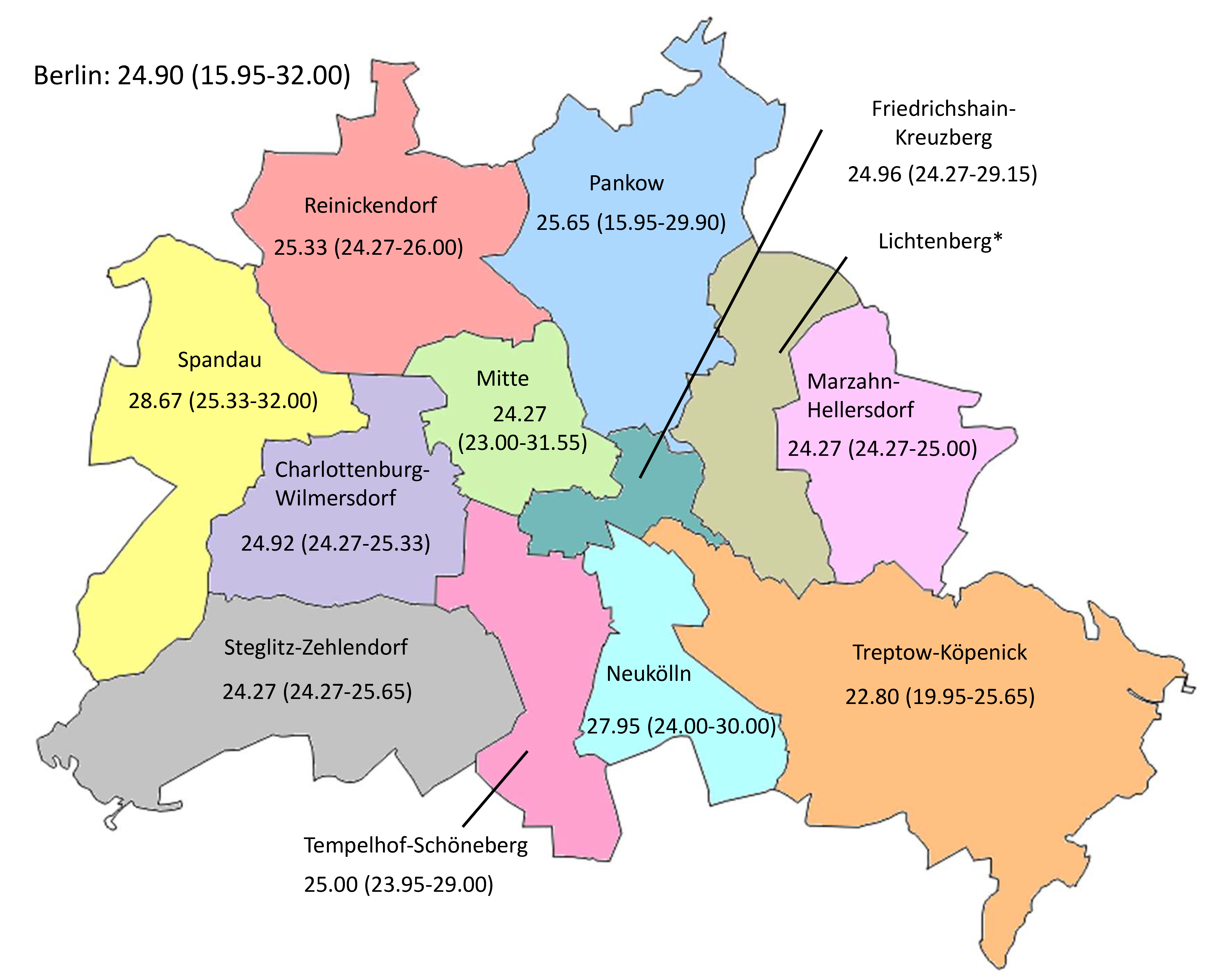


Note: * not calculable due to n = 1.

Additional file 2: Price of LNG medications (n = 160) by district, median price (minimum price - maximum price)


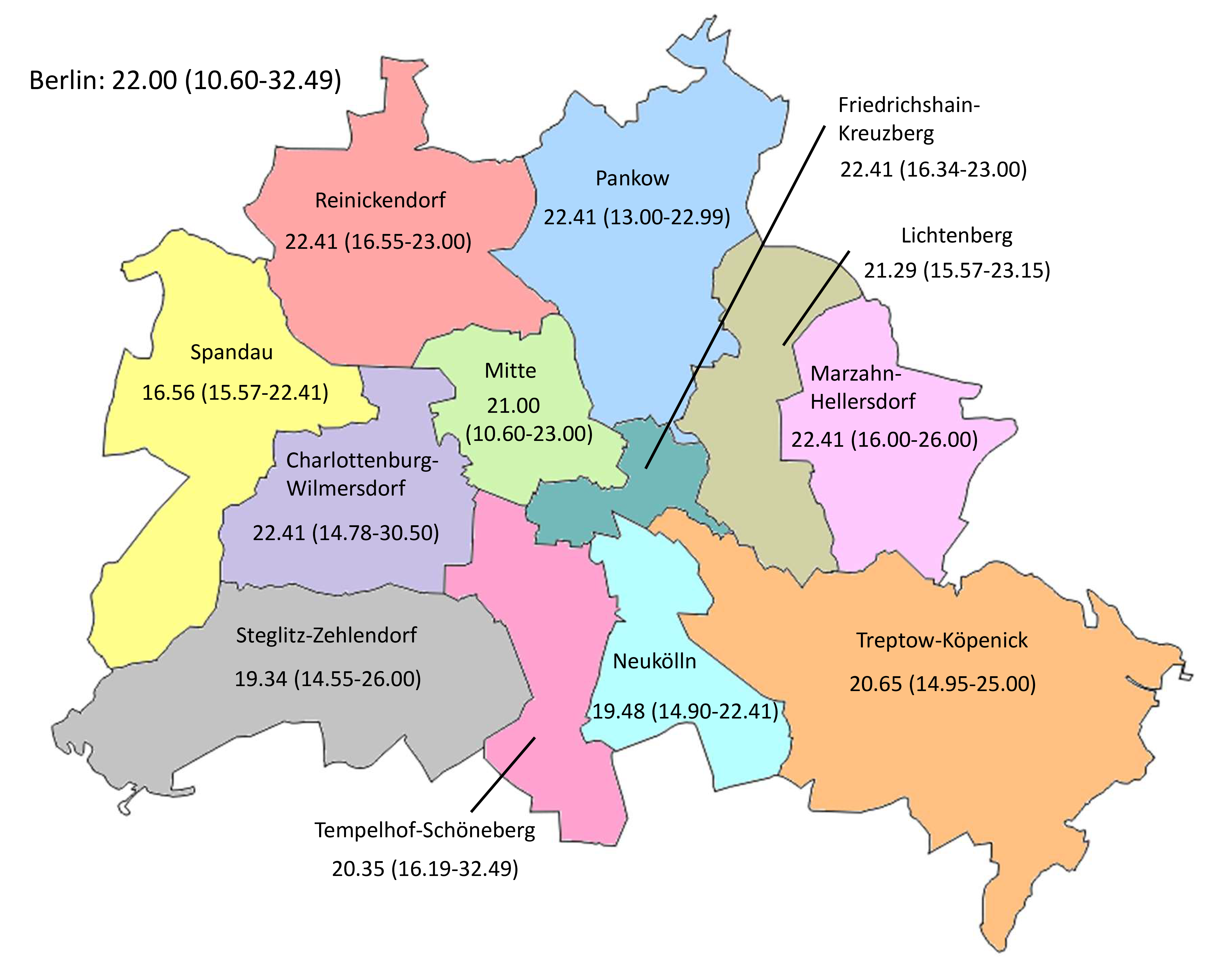

Supplement: Supplementary file 2 — Additional file 2. Price of UPA medications (n = 241) by district, median price (minimum price - maximum price). Price of ellaOne® (n = 224) by district, median price (minimum price - maximum price). Price of UPA generics (n = 50) by district, median price (minimum price - maximum price). Price of LNG medications (n = 160) by district, median price (minimum price - maximum price). [file 40545_2023_565_MOESM2_ESM.docx]
